# Magnetic Resonance Image Guided Focused Ultrasound Thalamotomy for Essential Tremor in a Patient with an Arteriovenous Malformation

**DOI:** 10.5334/tohm.1210

**Published:** 2026-05-27

**Authors:** Tomaima Yousef, Elma A. Chowdhury, Piush Sarkar, Hooman Azmi

**Affiliations:** 1 Department of Neurosurgery, Hackensack Meridian School of Medicine, Nutley, NJ, US; 2 Department of Neurosurgery, Hackensack University Medical Center, Hackensack, NJ, US

**Keywords:** focused ultrasound, thalamotomy, arteriovenous malformation, essential tremor, case report

## Abstract

**Background:**

Magnetic Resonance Guided Focused Ultrasound Thalamotomy (MRgFUS) is a non-surgical treatment option for medically refractory Essential Tremor (ET) or tremor dominant Parkinson’s Disease.

**Case Report:**

A 75-year-old left-handed man with medically refractory essential tremor presented for MRgFUS evaluation. During workup, contrast-enhanced brain MRI revealed an incidental right frontal arteriovenous malformation. After discussion of management options, the patient elected not to pursue treatment of the newly diagnosed AVM and proceeded with tremor-focused therapy.

**Discussion:**

It is possible to effectively utilize MRgFUS for treatment of tremors in carefully selected patients with cerebral AVMs by designating the malformation as a no pass zone.

## Introduction

Magnetic resonance guided focused ultrasound (MRgFUS) thalamotomy is a nonincisional technique used to treat medication-refractory Essential Tremor (ET) or tremor-dominant Parkinson’s Disease (TDPD). MRgFUS has been extensively studied for the treatment of symptomatic uterine fibroids, prostate cancer, and continues expanding clinically [4,5]. This technique uses high intensity ultrasound (HIFU) waves to coalesce on a target of interest to create a thermal lesion [6]. The procedure is monitored by magnetic resonance imaging (MRI) to allow for highly accurate real-time visualization of temperature changes of the brain tissue. Although other effective treatments for ET exist, including deep brain stimulation (DBS) and stereotactic radiosurgery with gamma knife, MRgFUS has proven to be a well-established alternative that provides real-time image guidance and immediate clinical feedback.

A limitation of MRgFUS, however, is its use in patients with MRI conditional implants. Other considerations when planning for this treatment include the need to create “no-pass zones” in the settings of prior craniotomies or in the presence of skull implanted devices, such as shunts [1]. Prior studies have illustrated the feasibility MRgFUS thalamotomy in patients with implanted IPGs [3], shunts [7], and prior craniotomies [8]; however, there is no literature demonstrating the feasibility of MRgFUS thalamotomy in a patient with an arteriovenous malformation (AVMs). Another complexity in patients with AVMs is the low but non-zero risk of coagulopathies and hemorrhagic complications with MRgFUS [9,10]. This is an especially prominent consideration in this patient with an AVM in whom the main concern is risk of hemorrhage. This report presents a case of successful MRgFUS thalamotomy in a patient with a right frontal AVM, with all the aforementioned elements taken into account.

### Illustrative Case

#### Clinical Presentation

**Figure 1:**
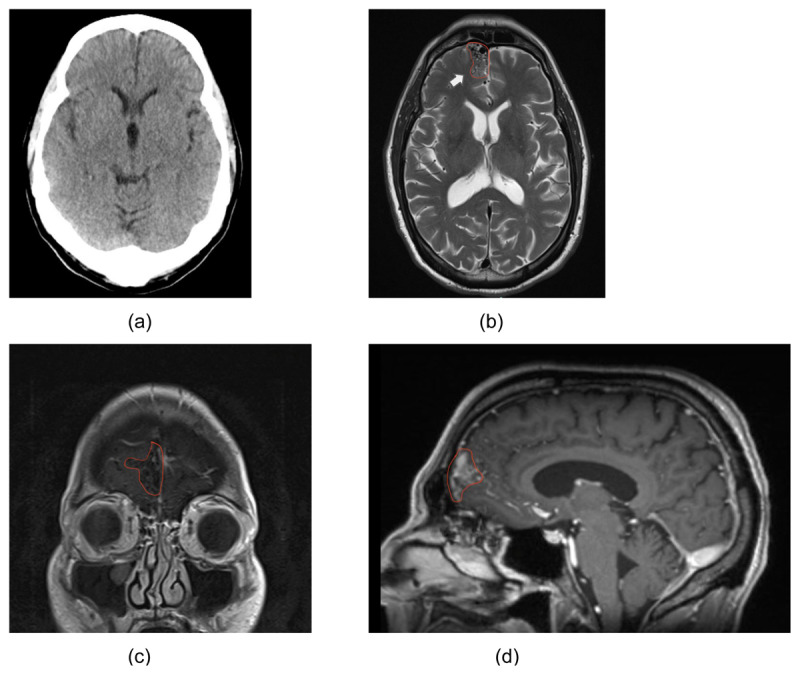
**(a)** CT-scan originally done during the work-up process. **(b)** T2-weighted Axial MRI demonstrating the AVM at the right frontal lobe. **(c)** T1-weighted sagittal MRI of the AVM at the right frontal lobe. **(d)** T1-weighed coronal MRI of the AVM at the right frontal lobe.

The patient is a 75-year-old male with a long history of asymmetrical essential tremor (more prominent in the left hand) that is refractory to medical therapy. His past medical history includes benign prostatic hyperplasia with urinary frequency, right renal cyst, and hepatic hemangioma. Medications included finasteride, primidone, propranolol, and tamsulosin. Family history is notable for kidney cancer. During the pre-treatment workup, the patient was found to have an incidental right-sided frontal AVM, characterized as Spetzler-Martin Grade 1 and Lawton-Young Grade 5 (Figure 1). The patient was evaluated for this finding, with discussion of management options including endovascular treatment, surgical resection and radiosurgery, and lifetime cumulative rupture risks. After the discussion with the patient, expectant surveillance was selected for the management of the AVM; subsequently, the patient elected to proceed with MRgFUS thalamotomy for his tremors. His tremors impaired his activities of daily living (ADLs) and writing despite high doses of medication. For neurological examination pre-procedural, the patient is alert and oriented to person, place, and time. Although he reported memory difficulty, cognitive testing showed no deficits. He demonstrated a forward head posture with resting head tremor and wide-based gait. Right upper extremity tremor was more prominent than the left. Motor strength was ⅘ bilaterally with no sensory deficits. CRST scoring pre-procedural was noted to be [2] on parts A-C (Figure 3).

To ensure feasibility, the procedure was simulated on the device console preoperatively. The AVM was marked as a “no-pass zone” to determine active element availability.

#### Intraoperative Findings

**Figure 2:**
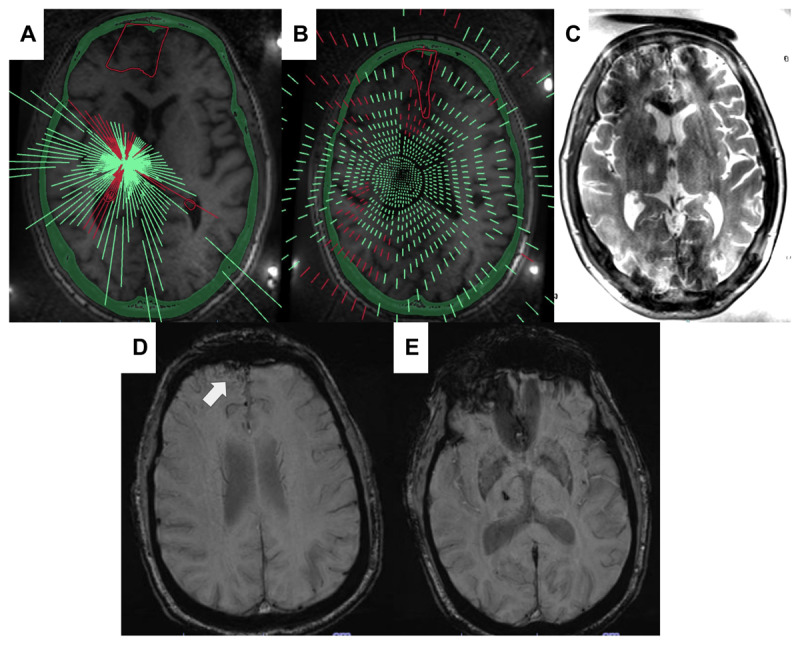
**A** and **B** depicts the procedural demonstration of transducer element summaries with the no pass zones of the AVM outlined in red. **C** depicts the subsequent postoperative T2-weighted axial MRI demonstrating the thalamotomy lesion in the right thalamus. Images **D** and **E** are post-procedural susceptibility weighted imaging demonstrating an unchanged right frontal AVM (D) and the right thalamotomy (E), with no additional evidence of hemorrhage.

After stereotactic frame placement, volumetric T1 imaging was obtained. The anterior and posterior commissures were identified, with a measured distance of 25.65 mm. Target coordinates were selected (X: 15, Y: 7.5, Z: 1.5). The AVM and associated vessels were designated as “no-pass zones” (Figure 2A–B). A total of 885 active elements remained with a skull density ratio (SDR) of 0.58. Table 1 summarizes sonication parameters. Two targets were successfully lesioned without adverse effects.

**Table 1:** The operative characteristics of the MRI-guided focused ultrasound procedures at the initial stage and the second stage at postoperative day five.


SONICATION NUMBER	TARGET COORDINATES			PROCEDURE STEP	SONICATION PARAMETERS		PRESCRIBED DOSE (J)	DELIVERED DOSE (J)	AVERAGE TEMPERATURE (°C)	MAXIMUM TEMPERATURE (°C)
	
	X	Y	Z		POWER (W)	TIME (s)				

1	15	7.5	1.5	align	192	12	2,200	2,217	43	44

2	15	7.5	1.5	align	290	12	3,300	3,316	43	44

3	15	7.5	1.5	align	336	12	3,850	3,881	44	49

4	15	7.5	1.5	align	335	14	4,550	4,099	47	52

5	15	8.5	1.5	verify	578	16	8,400	8,458	50	51

6	15	8.5	1.5	verify	605	7	12,350	43,335	40	39

7	15	8.5	1.5	verify	635	20	12,350	12,289	52	55

8	15	8.5	1.5	treat	818	36	28,798	28,830	56	57

9	15	9.5	3	treat	817	39	31,339	31,343	52	55


#### Postoperative Clinical and Radiographic Outcomes

**Figure 3:**
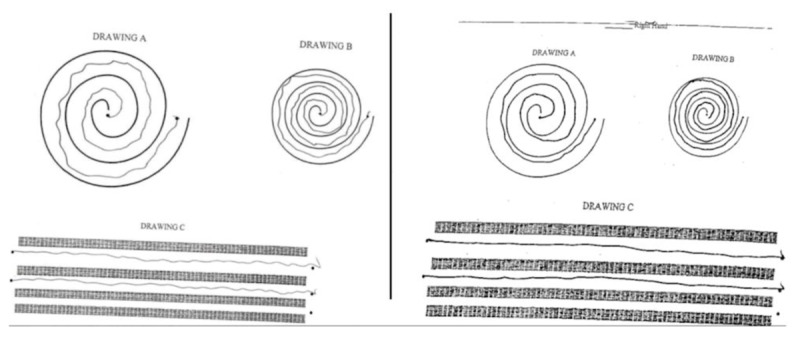
The left panel demonstrates the preoperative evaluation of the patient’s tremors. The right panel demonstrates the patient’s one-year postoperative display improvement with decreased intrusions.

Following MRgFUS, the patient’s CRST score improved on the post-ablation Archimedes spiral test; on CRST scoring, the patient scored 0 on Part A, 1 on Part B, and 0 on Part C (Figure 3). The patient reported the ability to perform his ADLs independently and rated himself “much improved” on the Patient Global Impression of Change (PGIC). The patient followed up with the neurosurgery team several times post-treatment. MRI at 3 months and 1 year showed expected thalamotomy changes with stable AVM appearance and no hemorrhage. At the eight month follow up with physical therapy, the team noted that the patient disclosed improvements in terms of his “shaving, writing, and eating” with his left hand. He does report some difficulty with cutting food and grabbing objects with his right hand, but discloses no pain after the procedure. In terms of posture, the patient remained to have a forward head with a stable resting tremor of the head, wide based stride and gait, and ⅘ strength in all of his extremities. Neurologic examination remained stable aside from tremor. In 2025, the patient was re-evaluated by physical medicine and rehabilitation therapy documented stable moderate right-hand intention tremor without left-sided tremor.

## Discussion

MRgFUS thalamotomy has emerged as an effective therapeutic option for the treatment of disabling tremor that interferes with activities of daily living in patients with medically refractory essential tremor and tremor-dominant Parkinson’s disease. Traditionally considered second-line to DBS, recent evidence challenges this paradigm. A systematic review by Giordano and colleagues published in 2020 found no statistically significant difference (p < 0.198) between MRgFUS and unilateral DBS in terms of tremor improvement [11]. At the same time, postoperative quality-of-life improvement was significantly greater following MRgFUS thalamotomy, suggesting that both approaches may be reasonable first-line options, with treatment selection guided by patient preference. Additionally, research that was recently published in 2026 [12] in the Journal of Neurosurgery Review by Sabet and colleagues found that DBS carries risks related to its invasive nature, including intracranial hemorrhage, which would be particularly undesirable in a patient with an arteriovenous malformation (AVM). Ultimately, this was the clinical scenario encountered in our patient, for whom the risks and benefits of DBS and MRgFUS were carefully discussed in the context of their unique anatomical considerations, leading to the decision to proceed with MRgFUS. Since FDA approval in 2016, MRgFUS has been shown to provide effective tremor control, with robust and durable improvements in tremor severity, functional disability, and quality of life. It is also associated with a favorable safety profile compared with other surgical options such as DBS or stereotactic radiosurgery, with adverse effects that are typically mild and transient.

The main concern when conducting MRgFUS in similar patient profiles who have AVMs is twofold. At the outset, there is the risk of causing the AVM to rupture. Secondly, there is the need to reduce the number of active elements to reduce the risk of rupture while simultaneously maintaining sufficient energy to create a successful lesion. This part of the discussion will primarily focus on the first concern, as the second involves dose-escalation balance of gradually increasing energy to cross therapeutic threshold. The first concern aforementioned is grounded in the formation of a hematoma due to MRgFUS, which is both a real and theoretical risk. Within the research conducted by Martínez-Fernández et al. [13] it was found that out of five hundred treated patients with Parkinson’s disease and no prior AVMs, two developed hematomas associated with cavitation during treatment. Even in the absence of AVMs, MRgFUS carries the risk of rupturing vessels in the brain parenchyma. Moreover, the conceptual understanding of rupture is grounded in the works demonstrated by Siu et al. [14] demonstrated in MRgFUS trials. In the research conducted in 2017, researchers created a gel phantom with two tubes of various diameters (0.76 mm and 3 mm) to mimic small vessels [14]. This model is comparable to the brain parenchyma, as small cerebral arteries are conventionally less than 2.5 mm in diameter. They discovered that as the sonications from the MRI machine intensified, thermal coagulation occurred within the tubes, ultimately leading to rupture. From a mechanistic standpoint, as the energy required to achieve therapeutic temperatures increased, so did the frequency of rupture in the model system, which reflects the aforementioned theoretical concern in our patient. However, the researchers acknowledged that there is a limited body of evidence specifically linking hematoma formation or rupture of AVMs in human trials to MRgFUS, underscoring the need for further investigation into this clinically relevant concern. They also noted the vastly different opinions of other researchers, who suggest that MRgFUS may, in fact, treat AVMs without precipitating rupture [15]. An important consideration, as demonstrated by Martínez-Fernández et al. [13], is a favorable SDR as a means to mitigate the risk of cavitation by improving heating efficiency, and thereby lowering the risk of intracranial hemorrhage. During pre-procedural planning, it was determined that our patient had a favorable SDR of 0.58 and, coupled with the lack of cavitation during the procedure, there was a decreased risk of hemorrhage. In any case, the potential for AVM rupture warrants consideration, as the current body of evidence does not support a definitive conclusion, thereby favoring a cautious approach and preserving clinical equipoise.

As for the second concern, through pre-procedural planning, the AVM was marked as a no pass zone and the elements overlying it were blocked- resulting in the exclusion of 224 transducers. Literature indicates that a minimum of 800 elements [16] is necessary to obtain therapeutic temperatures. For the case of our patient, there were a total of 885 active elements indicating an adequate number for lesioning during the procedure. In addition to the number of employed transducers, there are several critical parameters involved in the process of creating a successful ablation in the thalamus for essential tremors: temperature, skull volume, and the skull density ratio [17,18]. Temperatures greater than 55°C are generally required to create a thermal lesion; although a temperature of above 50°C for over ten seconds can also be lesional [18]. Despite pre-procedural blocking resulting in decreased employed transducers, we were able to create a successful lesion in our patient by reaching adequate temperatures.

With the success of the procedure, it is worth noting that the novelty of the case lies in the anatomical challenges of the surgical field in the context of the AVM. There has been prior literature establishing the procedural framework [19,20] for performing HIFU for essential tremors and even creating no-pass zones for different anomalies [18]. The positive outcome of this procedure expands the application of MRgFUS, but does not generalize its safety onto all patients, as individual patients may have idiosyncratic surgical risks.

## Conclusion

This case demonstrates successful MRgFUS thalamotomy in a patient with essential tremor and an incidental AVM. The procedure achieved durable tremor improvement without hemorrhagic complications, supporting feasibility in carefully selected patients with vascular malformations.
